# Agreement between CT-Angiography and Digital Subtraction Angiography in Predicting Angiographic Vasospasm in Patients with Subarachnoid Hemorrhage

**DOI:** 10.3390/jcm13133743

**Published:** 2024-06-26

**Authors:** Miriam M. Moser, Leon Gramss, Wolfgang Marik, Michael Weber, Dorian Hirschmann, Wei-Te Wang, Philippe Dodier, Gregor Kasprian, Gerhard Bavinzski, Karl Rössler, Arthur Hosmann

**Affiliations:** 1 Department of Neurosurgery, Medical University of Vienna, 1090 Vienna, Austria; miriam.moser@meduniwien.ac.at (M.M.M.); leongramss@gmail.com (L.G.); dorian.hirschmann@meduniwien.ac.at (D.H.); wei-te.wang@meduniwien.ac.at (W.-T.W.); philippe.dodier@meduniwien.ac.at (P.D.); gerhard.bavinzski@meduniwien.ac.at (G.B.); karl.roessler@medunwien.ac.at (K.R.); 2 Department of Neuroradiology and Musculoskeletal Radiology, Medical University of Vienna, 1090 Vienna, Austria; wolfgang.marik@meduniwien.ac.at (W.M.); michael.weber@meduniwien.ac.at (M.W.); gregor.kasprian@meduniwien.ac.at (G.K.)

**Keywords:** subarachnoid hemorrhage, CT-angiography, digital subtraction angiography, cerebral vasospasm, delayed cerebral ischemia

## Abstract

**Background/Objectives:** Digital subtraction angiography (DSA) is the gold standard in the diagnosis of cerebral vasospasm, frequently observed after subarachnoid hemorrhage (SAH). However, less-invasive methods, such as computed tomography angiography (CTA), may be equally accurate. To further clarify comparability, this study evaluated the reliability of CTA in detecting cerebral vasospasm. **Methods**: This retrospective study included 51 patients with SAH who underwent both CTA and DSA within 24 h. The smallest diameter of the proximal cerebral arterial segments was measured in both modalities at admission and during the vasospasm period. The mean difference in diameter, the intraclass correlation coefficient (ICC) of CTA and DSA, the difference in grade of vasospasm and sensitivity, the specificity and the positive predictive value (PPV) for CTA were calculated. **Results**: A total of 872 arterial segments were investigated. At time of admission, arterial diameters were significantly smaller on CTA compared to DSA in all segments (−0.26 ± 0.12 mm; *p* < 0.05). At time of suspected vasospasm (day 9 ± 5), these differences remained significant only for the M1 segment (−0.18 ± 0.37 mm, *p* = 0.02), the P1 segment (−0.13 ± 0.24 mm, *p* = 0.04) and the basilar artery (−0.20 ± 0.37 mm, *p* = 0.0.04). The ICC between CTA and DSA was good (0.5–0.8). The sensitivity of CTA for predicting angiographic vasospasm was 99%, the specificity was 50% and the PPV was 92%. **Conclusions**: Arterial diameters measured on CTA may underestimate the arterial caliber observed in DSA; however, these absolute differences were minor. Importantly, vessel diameter alone does not fully reflect malperfusion, requiring additional imaging techniques such as CT perfusion.

## 1. Introduction

Cerebral vasospasm is a common and potentially life-threatening complication after subarachnoid hemorrhage (SAH). Angiographic visible vasospasm occurs in 40–70% of patients after SAH, which might result in reduced cerebral perfusion, resulting in devastating ischemic stroke and secondary neurological damage [1].

Digital subtraction angiography (DSA) remains the gold standard for the diagnosis of cerebral vasospasm due to its high sensitivity and specificity [2,3,4,5,6,7]. Computed tomography angiography (CTA) is considered a suitable non-invasive method for the diagnosis and quantification of vasospasm, with a reported sensitivity of about 88% and a specificity of about 84% [8].

Cerebral vasospasm and delayed cerebral ischemia often occur at similar time points in patients after SAH [9] and result in bad neurological outcomes and death [10]. The pathogenesis of delayed cerebral ischemia remains a matter of debate and results from multifactorial genesis [9,11]. However, radiographical signs of vasospasm often correlate with DCI, which is therefore the most obvious sign, and therefore have a significant impact on further clinical management [12]. This is of particular significance in the context of neurosurgery intensive care, where patients are frequently deeply sedated. Thereby, neurological deterioration may not be clinically detected. Consequently, the CTA represents a valuable non-invasive method for the detection of vasospasm, because especially in critically ill patients are non-invasive methods preferred.

However, previous studies have demonstrated a discrepancy in vessel diameters between CTA and DSA [10,13,14,15,16,17]. Some authors, for example, reported good correlation between CTA and DSA measurements and high sensitivity and specificity, especially in severe vasospasm and with no vasospasm [15], while others described high sensitivity and specificity, especially in mild and moderate vasospasm [16]. Another study reported a higher correlation coefficient between CTA and DSA in the vasospasm period than at admission [18].

We therefore aimed to further investigate the reliability of CTA in predicting angiographic cerebral vasospasm in patients with SAH.

## 2. Materials and Methods

### 2.1. Population

In this retrospective study, 51 patients (36 female, 15 male) suffering from SAH that underwent both CTA and DSA within 24 h within the first 21 days after their bleeding were included at the Department of Neurosurgery of the Medical University of Vienna between 2010 and 2021. This study was approved by the Ethics Committee of the Medical University of Vienna before its initiation (EK-Nr. 1134/2022). All study procedures adhered to the principles of the Declaration of Helsinki.

According to our institutional protocol for SAH patients, long-term sedation was performed primarily in poor-grade patients with decreased levels of consciousness (Glasgow Coma Scale < 9) at admission, global cerebral edema with effacement of basal cisterns and hemispheric sulci, and initially elevated intracranial pressure for maximum cerebral protection. Initially, continuous propofol and remifentanil infusion was used, which was switched to midazolam (up to 20 mg/h) and sufentanil (up to 0.25 μg/kg/min) after 3–5 days. In case of inadequate sedation, ketamine (up to 200 mg/h) and metohexital (up to 1 mg/kg/h) were added in ascending order.

Nimodipine was administered orally every 4 h at a dose of 60 mg in 30 patients or as continuous intravenous infusion at a maximum dose of 2 mg/h in 10 patients. In 10 patients (treated between 2010 and 2013), nimodipine therapy information was not available.

### 2.2. Radiological Assessment

Measurement of flow velocities within both main middle cerebral artery trunks using TCD was performed at least once per day. Flow velocities > 120 cm/s were considered as increased. At new onset of focal neurological deficits and/or deterioration of consciousness in the awake patients or increased TCD flow velocities in the sedated patients, CT and CT-angiography scans were performed. In cases of severe cardiac pathology, we increased the HU from 130 to 200 before starting the CTA to increase intracranial contrast by delayed starting. We therefore tried to overcome the problem with poor cardiac afterload.

CTA typically employs a relatively low dose of contrast agent, in contrast to other imaging modalities, with 50 mL of Iomeron 400 being the standard dose. This allows for the examination of patients with impaired renal function, although the risk of nephrotoxicity cannot be entirely eliminated. Nevertheless, over the past decade, we have not encountered any cases of acute kidney injury resulting from a single CTA examination.

CTA was performed on the following scanners: a Siemens Somatom Edge Plus, a Siemens Somatom Drive, a Siemens Somatom Definition Edge, a Siemens Somatom Definition AS+, a Siemens Sensation 64, a Siemens Sensation Cardiac 64, and a Siemens Sensation 16 or Philips MX 16-slice. The diversity of the CT scanners utilized can be attributed to the acute setting in which the measurements were performed. Consequently, the first available CT scanner was approached.

All digital subtraction angiography procedures were performed by endovascular-trained neurosurgeons. Following femoral artery puncture, a 6F arterial sheath was introduced and a guiding catheter navigated to the supra-aortic arteries of interest, i.e., internal carotid arteries and vertebral arteries. Biplane angiography was performed to assess degree and distribution of arterial vasospasm. The DSA procedures were performed on a Siemens Axiom Artis dBA and on a Siemens artis icono.

The smallest diameter of the main cerebral arterial vessels in millimeters (mm) was independently measured in the CTA scans and DSA by two investigators (M.M.M. and L.G.). Both readers were trained on orthogonal vessel measurements using 10 real-world data sets under the supervision of an experienced radiologist (M.W., >12a experience) before starting with the study data sets.

The vessel diameter was determined in the following arterial segments: both sides of the A1 segment of the proximal anterior cerebri arteries (ACA), the M1 segment of the middle cerebral arteries (MCA), the P1 segment of the posterior cerebral arteries (PCA), the intradural part of the internal carotid arteries (ICA), the V4 segment of the vertebral arteries (VA) and the non-paired basilar artery (BA). Measurements were taken at the narrowest point of each of the above vessel segments in the DSA using the anterior–posterior projections for the ACA, MCA, PCA, BA and VA and the lateral projection for the ICA. For the vessels located to the right and the left of the circulus arteriosus (i.e., M1, A1, ICA, P1, and VA), the mean value of the diameter was calculated for further calculations.

Using a 3D multislice CTA data set, the vessel segments of interest were reconstructed parallel to the vessel lumen. The location corresponding to the selected location in the DSA was measured accordingly in the CTA images. To minimize inter-observer variability, the mean values of measurements from the two independent investigators were calculated for further analysis.

A recent review of the literature has demonstrated poor repeatability of vasospasm grading in CTA between observers, likely because of different grading systems and reference parameters [19]. Therefore, we decided on the following approach:

The assessment of both imaging modalities was divided into vessel diameters at admission (within 3 days after the bleeding) and diameters during the vasospasm period (3–21 days after the ictus). Vasospasm was graded for each arterial segment in comparison to initial DSA at admission as mild for a vessel diameter of 60–99% of the physiological lumen, moderate for a vessel diameter of 30–59%, and severe for a vessel diameter less than 30% of the physiological lumen.

### 2.3. Statistical Analysis

Patients’ demographics were calculated as the total number and percent of the study population for discrete parameters and mean and standard deviation for continuous parameters. Following an assessment of the distribution for normality using the Kolmogorov–Smirnov test, differences in vessel diameter were calculated using a paired *t*-test with 95% confidence intervals and a two-sided alpha of 0.05. The intraclass correlation coefficient (ICC) was calculated to assess the correlation of CTA and DSA at time of admission and time of suspected vasospasm. No correction for multiple testing was performed due to the exploratory design of the study. The mean and standard deviation between the two independent investigators were calculated. Bland–Altman plots show concordance in vessel segment diameter in CTA and DSA at admission and at time of suspected vasospasm. W.M. supervised the statistical analyses.

## 3. Results

### 3.1. Population

In 51 patients with SAH, a total of 872 arterial (paired) segments were investigated. The average age of patients was 48 ± 11 years, including 36 women (70.6%). Patients presented with a mean Hunt and Hess score of 3 ± 1 at admission. All patients were deeply sedated, with the exception of nine patients. In two cases, the state of wakefulness was not documented. The mean maximum reported transcranial Doppler velocity of the MCA was 180 cm/s ± 36 cm/s. Detailed patient characteristics are shown in Table 1.

CTA and DSA at admission were performed at a mean of 1 ± 3 days after the bleeding event. CTA and DSA at time of suspected vasospasm were performed 9 ± 5 days after the bleeding event. The mean time difference between CTA scan and DSA was 7 h 49 min (minimum 25 min, maximum 23 h 30 min) at admission, while at the time of suspected vasospasm it was 8 h 18 min (minimum 1 h 18 min, maximum 23 h 46 min).

The inter-investigator mean standard deviation of CTA measurements was ±0.2 mm for the ICA, the M1 segment, the BA and the P1 segment, ±0.1 mm for the A1 segment, and ±0.3 mm for the VA. The inter-investigator mean standard deviation of DSA measurements was ±0.2 mm for the ICA and the M1 segment, ±0.1 mm for the A1 segment and the BA, and ±0.3 mm for the P1 segment and the VA.

### 3.2. Radiological Assessment

#### 3.2.1. At Admission

Concomitant CTA and DSA was available in all included patients at admission; 566 (paired) vessel segments were measured.

**Figure 1:**
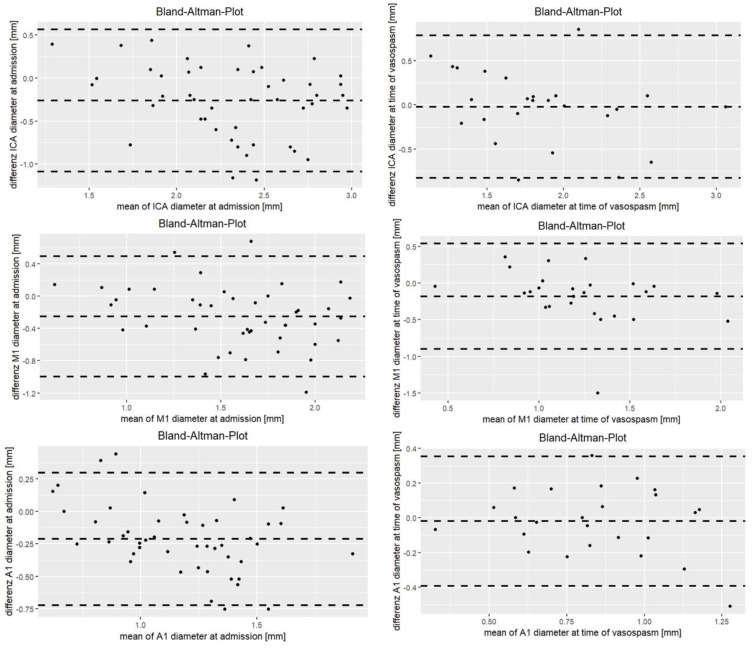
Bland–Altman plot of the anterior circulation at baseline and time of suspected vasospasm.

**Figure 2:**
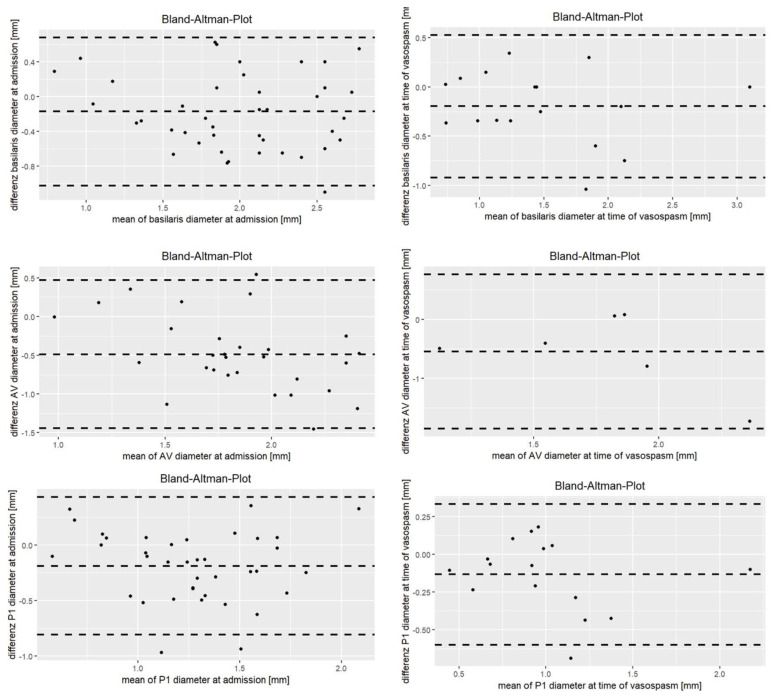
Bland–Altman plot of the posterior circulation at baseline and time of suspected vasospasm.

Overall, there was a significant correlation in vessel diameters between CTA (1.54 ± 0.42 mm) and DSA (1.81 ± 0.45 mm; r = 0.95; *p* < 0.001). The ICC at time of admission was between 0.51 and 0.65 in all segments, with the exception of the VA, where ICC was only 0.23 (Table 2). The concordance of vessel segment diameters between CTA and DSA at admission is illustrated in Figure 1 and Figure 2.

**Figure 3:**
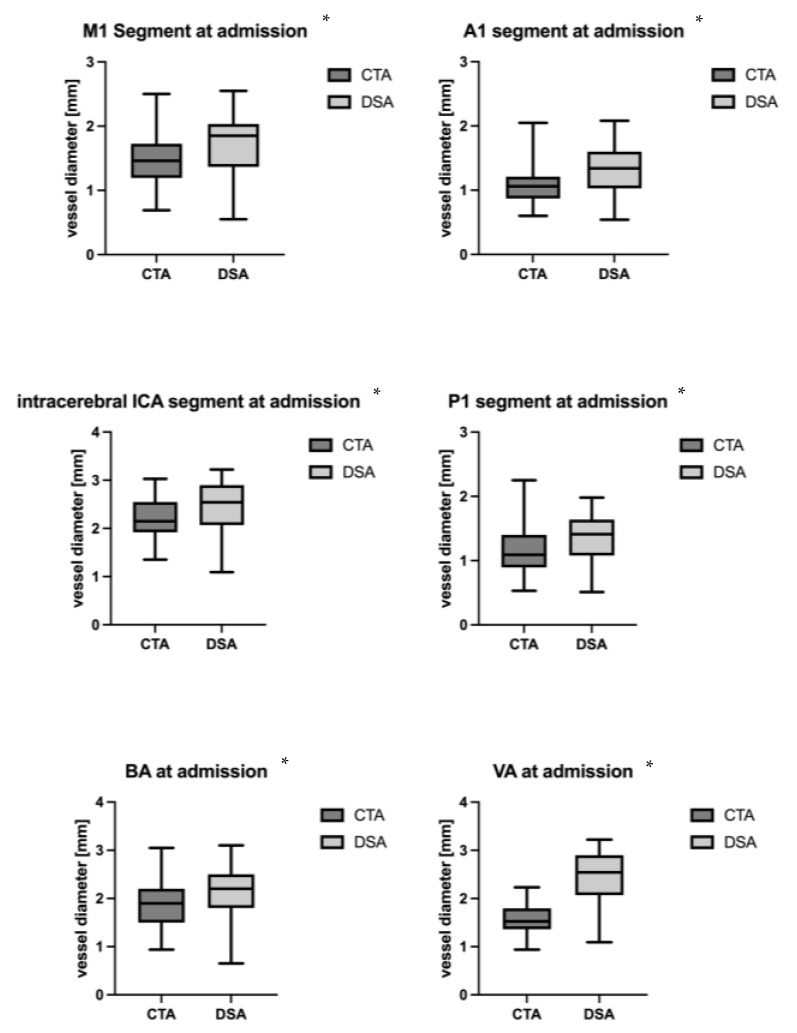
Difference in diameter at baseline in CTA and DSA in different segments. * Statistically significant.

However, there was a statistically significant difference in vessel diameter, showing a significant smaller diameter in CTA compared to DSA in all segments (Table 2, Figure 3). The mean difference in vessel diameter between CTA and DSA was −0.26 ± 0.12 mm, showing the lowest difference in the BA (−0.17 ± 0.43 mm) and P1 segment (−0.19 ± 0.32 mm) and the highest discrepancy in the VA (−0.49).

#### 3.2.2. Vasospasm Period

CTA and DSA scans during the vasospasm period were available in 28 of the included patients (55%). The DSA was performed at a mean of 9 ± 5 days after the bleeding; 306 (paired) vessel segments were measured.

The ICC at time of suspected vasospasm was between 0.53 and 0.80, with the exception of the VA, where the ICC was only 0.16 (Table 2). The concordance of vessel segment diameters between CTA and DSA during vasospasm is illustrated in Figure 1 and Figure 2.

**Figure 4:**
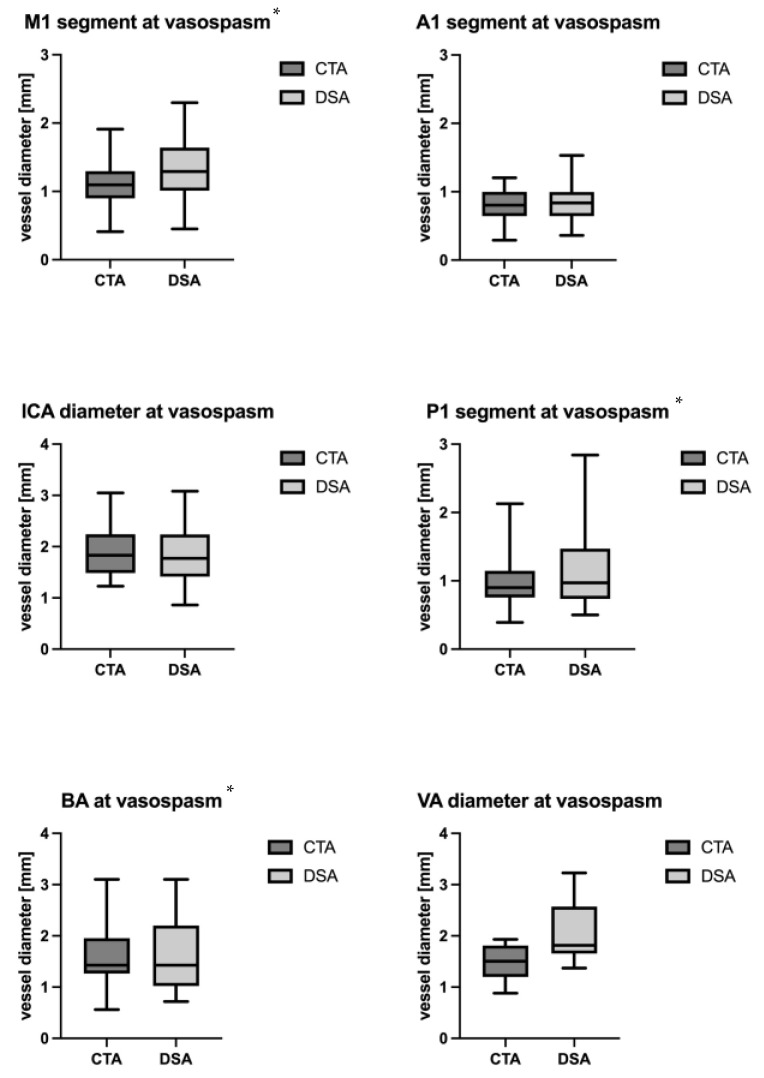
Difference in diameter at time of suspected vasospasm in CTA and DSA in different segments. * Statistically significant.

During the vasospasm period, a mean difference of −0.18 ± 0.20 mm in vessel diameter was observed between the CTA and the DSA. Specifically, significant differences were noted in the M1 segment (−0.18 ± 0.37 mm, *p* = 0.02), the P1 segment (−0.13 ± 0.24 mm, *p* = 0.04) and the BA (−0.20 ± 0.37 mm, *p* = 0.0.04). However, no significant differences in diameter were observed in the A1 segment (−0.02 ± 0.41 mm; *p* = 0.62), the ICA (−0.02 ± 0.41 mm; *p* = 0.79) and the VA (−0.55 ± 0.67 mm; *p* = 0.1) (Table 2, Figure 4).

Regarding diagnostic performance, the sensitivity of CTA to accurately detect and classify angiographic vasospasm in our cohort was 99%. However, the specificity was 50%, and the positive predictive value was 92%.

There was no significant difference in vasospasm grading between DSA and CTA in the ICA (*p* = 0.95), A1 segment (*p* = 0.75), VA (*p* = 0.12), BA (*p* = 0.06), and P1 segment (*p* = 0.06). However, vasospasm grading was notably worse in CTA compared to DSA for the M1 segment (*p* = 0.008) (Table 3).

## 4. Discussion

We evaluated the accuracy of CTA measurements of cerebral vessels in detecting angiographic vasospasm after SAH by comparing vessel diameter in CTA and DSA on the same day. We identified a strong correlation between these two modalities. Upon admission, significant differences in vessel diameters between CTA and DSA were observed in all segments. However, these observed differences were less than 1 mm and therefore likely clinically negligible. During the vasospasm period, discrepancies in diameter between the two imaging modalities were evident primarily in the M1 segments, VA and BA. Once more, when considering the absolute values, these differences were minimal and thus probably not clinically relevant.

CTA had a sensitivity of 99% and a positive predictive value of 92% to detect vasospasm. Consequently, our results of the small absolute difference in vessel diameters and the good correlation are emphasized. Nevertheless, the specificity observed in our cohort was only 50%, which is in concordance with but slightly lower than the previously reported specificity of about 60% [8].

The current gold standard for diagnosing cerebral vasospasm remains DSA [7]. However, this approach is both invasive and time-consuming and requires the patient to remain in a horizontal position for an extended period, which can pose risks of intracranial hypertension and cerebral malperfusion. Nonetheless, once severe cerebral vasospasm is diagnosed, therapeutic spasmolysis or angioplasty can be performed during the same procedure [20,21]. In contrast, CTA is a non-invasive, readily accessible modality and may therefore be preferred at critical time points. However, even this non-invasive procedure can have detrimental effects on cerebral perfusion in critically ill patients [22]. In addition to CTA, transcranial Doppler sonography represents another non-invasive method that is readily accessible. This method has been reported to demonstrate a sensitivity of 80–90% and a specificity of 50%. Thus, this approach exhibits a slightly lower sensitivity than CTA [23,24]. Additionally, the effectiveness of this approach is dependent on the skill and experience of the operator [23,24]. Consequently, this study focused on CTA only. Previous studies have already compared CTA and DSA, but currently there is no consensus in the literature whether CTA measurements tend to over- or underestimate vessel diameters compared to DSA [13,14,15,16,17,18,25].

In patients with intracranial arterial stenosis, Niu et al. [25] found an overestimation of stenosis in the CTA. Anderson et al. [15] analyzed the correlation between DSA and CTA measurements and the sensitivity and specificity of CTA in the grading of vasospasm after SAH (none, mild, moderate, severe) in a prospective study. In severe or no vasospasm, there was a 92–100% agreement, while in moderate or mild vasospasm, only a 57–64% agreement was observed [15]. Thus, their findings indicated a high accuracy of CTA in the detection of severe vasospasm and no vasospasm, but lower accuracy for mild and moderate cases [15]. In contrast, Joo et al. [16] described high sensitivity, specificity, and accuracy in mild and moderate vasospasm, but not with no spasm or severe vasospasm. Our results are in line with those of Joo et al. [16] and indicated similar frequencies of mild and moderate vasospasm in CTA and DSA, but ‘no vasospasm’ was defined more often in DSA than in CTA. The variability of results in the literature may be attributed to different grading systems and reference parameters, as published in a recent review [19].

Kerkeni et al. [18] also compared CTA and DSA at baseline and at the time of vasospasm and focused on correlation. The correlation coefficient at admission was 0.64 and increased to 0.84 in the vasospasm period [18]. Similarly, in our study, the ICC was much higher at time of vasospasm than at admission. Overall, the results indicate at least moderate to good reliability for all segments, with exception of the VA, which demonstrated poor reliability at both time points.

Moreover, our study revealed only minimal differences in absolute values between CTA and DSA at admission. Given the number of different CT devices used in this study, the observed consistency across different companies is indicative of a high degree of comparability of results. The absolute difference between CTA and DSA did not exceed 0.5 mm in any case. At the time of suspected vasospasm, the differences between the measurements taken from CTA versus DSA showed significant changes (although still relatively minor in absolute terms) in the M1 segments, the right VA and the BA.

Additionally, the size of the arteries may also influence the accuracy of CTA measurements. Ferguson et al. [14], for instance, compared vessel diameters in DSA and CTA and found a significant correlation of the two modalities, but CTA generally underestimated the diameter of larger arteries and overestimated the diameter of small arteries, i.e., the more peripheral segments (A2, M2, P2, etc.) as defined by radiological classification. We only examined proximal vessels due to better comparability [18], and did observe smaller differences in diameter during periods of vasospasm, therefore supporting these results. Finally, differences in diameter and ICC in the vertebral artery exhibited considerable variability, and any changes in these segments may have to be interpreted with caution.

CTA tends to slightly underestimate arterial diameters compared to DSA. Although the differences are statistically significant, they are minor in absolute terms. Clinicians should be aware of this tendency, particularly in critical segments such as the M1, P1, and basilar artery, especially during the vasospasm period. Given the observed discrepancies, interpreting vessel diameters from CTA in these specific segments should be carried out with caution.

However, vessel diameter alone does not provide a complete picture of cerebral perfusion and malperfusion. Factors contributing to poor neurological outcomes, such as DCI, involve complex mechanisms beyond mere vessel narrowing, including microcirculatory constriction and cortical spreading depression. The pathogenesis of DCI is multifactorial and includes both macroscopic and microscopic components [9,11]. Relying solely on macroscopic vessel diameter measurements may overlook critical pathophysiological processes. To accurately diagnose and manage DCI, additional imaging modalities, such as CT perfusion imaging, are necessary. These techniques can offer more detailed insights into cerebral blood flow and perfusion deficits that are not detectable through diameter measurement alone. This approach will likely improve the accuracy of diagnosing vasospasm and DCI, leading to better-informed clinical decisions. Early and accurate detection of DCI through combined imaging could enable earlier intervention, potentially reducing the risk of long-term neurological deficits and improving overall patient outcomes. However, further prospective randomized trials are needed to verify this hypothesis [26,27,28]. Additionally, this combined approach may help avoid unnecessary invasive procedures such as DSA, thereby reducing patient risk and healthcare costs.

## 5. Conclusions

Arterial diameters measured on CTA may underestimate the arterial width observed in DSA. However, the observed differences were minor in absolute terms. At the time of vasospasm, diameter discrepancies were even smaller than in non-spastic vessels upon admission, and the correlation between the modalities was higher. Significant differences in diameter were noted, particularly in the M1 segments, VA and BA in the vasospasm period. Therefore, interpretation of vessel diameters in these segments in the CTA should be approached with caution. Moreover, cerebral malperfusion cannot be evaluated based solely on vessel diameter. Advanced imaging techniques such as CT-perfusion are needed to assess clinically relevant cerebral vasospasm in DCI.

## 6. Limitations

The limitations of this study are (1) its retrospective design and (2) the relatively small sample size. (3) The available data decreased from the baseline time point to the time of suspected vasospasm. (4) DSA during suspected vasospasm was primarily conducted on the arterial territory where vasospasm was presumed, resulting in incomplete visualization of all segments in those patients. (5) We only included the proximal segments of the cerebral blood vessels; due to difficulty in comparing data in more peripheral segments, it is not possible to draw any meaningful conclusions in this regard.

## Figures and Tables

**Table 1:** Patients’ demographics. * mean ± standard deviation; SAH = subarachnoid hemorrhage.

Age [years]		48 ± 11 *
sex	Female	36
	Male	15
Hunt and Hess Grade		3 ± 1 *
Type of SAH	Aneurysmal subarachnoid hemorrhage	49
	Prepontine subarachnoid hemorrhage	2
Ruptured aneurysm	- Middle cerebral artery	9
	- Anterior cerebral artery	0
	- Anterior communicating artery	19
	- Internal carotid artery	3
	- Basilar artery	2
	- Posterior communicating artery	6
	- Posterior inferior cerebellar artery	3
	- Anterior choroidal artery	3
	- Pericallosal artery	2
	- Superior cerebellar artery	1
	- No aneurysm	2
Type of intervention	Coiling	15
	Clipping	31
	Coiling and clipping	1
	No intervention	4
Timing of intervention (days after SAH)		1 ± 3 *
Sedation	Deeply sedated	40
	Awake	9
	State of wakefulness not documented	2
Mean maximum transcranial Doppler velocity [cm/s]		180 ± 36

**Table 2:** Difference in diameter between CTA and DSA and Pearson correlation at baseline and time of suspected vasospasm. Values are shown as mean ± standard deviation. M1 = proximal segment of the medial cerebral artery, A1 = proximal segment of the anterior cerebral artery, P1 = proximal segment of the posterior cerebral artery, VA = vertebral artery, BA = basilar artery, CTA = computed tomography angiography, DSA = digital subtraction angiography, SD = standard deviation.

Segment	ICA	M1	A1	VA	BA	P1
At time of admission						
CTA diameter [mm]	2.2 ± 0.41	1.48 ± 0.39	1.09 ± 0.29	1.57 ± 0.35	1.89 ± 0.51	1.16 ± 0.35
DSA diameter [mm]	2.45 ± 0.51	1.71 ± 0.47	1.28 ± 0.37	2.09 ± 0.52	2.08 ± 0.56	1.34 ± 0.40
Difference CTA—DSA [mm]	−0.26 ± 0.42 (95% CI −0.39; −0.14) *p* < 0.001	−0.25 (±0.38) (95% CI 0.37; −0.13) *p* < 0.001	−0.21 ± 0.26 (95% CI −0.29; −0.13) *p* < 0.001	−0.49 (±0.49) (95% CI −0.67; −0.30) *p* < 0.001	−0.17 (±0.43) (95% CI −0.31; −0.04) *p* = 0.01	−0.19 (±0.32) (95% CI −0.29; −0.09) *p* < 0.001
Intraclass correlation coefficient	0.51 (95% CI 0.18, 0.72)	0.53 (95% CI 0.18, 0.74)	0.55 (95% CI 0.12, 0.77	0.23 (95% CI −0.09, 0.54)	0.65 (95% CI 0.42, 0.80)	0.58 (95% CI 0.24, 0.77)
At time of suspected vasospasm						
CTA diameter [mm] mean ± SD	1.89 ± 0.47	1.12 ± 0.33	0.82 ± 0.22	1.48 ± 0.33	1.56 ± 0.58	0.96 ± 0.35
DSA diameter [mm] mean ± SD	1.85 ± 0.57	1.33 ± 0.45	0.83 ± 0.28	2.05 ± 0.65	1.58 ± 0.67	1.17 ± 0.61
Difference betweeen CTA and DSA [mm]	−0.02 ± 0.41 (95% CI −0.19; 0.15) *p* = 0.79	−0.18 ± 0.37 (95% CI −0.33; −0.03) *p* = 0.02	−0.02 ± 0.41 (95% CI −0.10; 0.06) *p* = 0.62	−0.55 ± 0.67 (95% CI −1.25; 0.15) *p* = 0.1	−0.20 ± 0.37 (95% CI −0.39; −0.01) *p* = 0.04	−0.13 ± 0.24 (95% CI −0.26; −0.01) *p* = 0.04
Intraclass correlation coefficient	0.69 (95% CI 0.41, 0.85)	0.53 (95% CI 0.18, 0.76)	0.72 (95% CI 0.46, 0.87)	0.16 (95% CI −0.33, 0.77)	0.80 (95% CI 0.51, 0.93)	0.80 (95% CI 0.48, 0.93)

**Table 3:** Grading of vasospasm: differences in vasospasm grading between CTA and DSA at time of suspected vasospasm.

VSP Grading at Time of Suspected Vasospasm in DSA and CTA					
		No Vasospasm	Mild	Moderate	Severe
ICA (*n* = 23)	DSA	9% (2)	61% (14)	30% (7)	0% (0)
	CTA	8.5% (2)	69.5% (16)	22% (5)	0% (0)
*p*-value	*p* = 0.95				
M1 (*n* = 24)	DSA	16% (4)	42% (10)	42% (10)	0% (0)
	CTA	4% (1)	42% (10)	54% (13)	0% (0)
*p*-value	*p* = 0.008				
A1 (*n* = 23)	DSA	4% (1)	46% (11)	46% (11)	0% (0)
	CTA	0% (0)	52% (12)	48% (11)	0% (0)
*p*-value	*p* = 0.75				
VA (*n* = 6)	DSA	33% (2)	50% (3)	16.6% (1)	0% (0)
	CTA	0% (0)	66.6% (4)	33.3% (2)	0% (0)
*p*-value	*p* = 0.12				
BA (*n* = 15)	DSA	26.6% (4)	33.3% (5)	40% (6)	0% (0)
	CTA	13.3% (2)	46.6% (7)	40% (6)	0% (0)
*p*-value	*p* = 0.06				
P1 (*n* = 14)	DSA	7% (1)	57% (8)	36% (5)	0% (0)
	CTA	12% (2)	50% (7)	43% (6)	7% (1)
*p*-value	*p* = 0.06				

## Data Availability

Data set available on request from the authors.
